# Development and psychometric evaluation of the epilepsy lifestyle questionnaire

**DOI:** 10.1371/journal.pone.0298773

**Published:** 2024-08-22

**Authors:** Masomeh Nadernejad, Abbas Shamsalinia, Reza Ghadimi, Payam Saadat, Alijan Ahmadi Ahangar, Kourosh Gharagozli, Fatemeh Ghaffari

**Affiliations:** 1 Department of Nursing, Babol University of Medical Sciences, Babol, Mazandaran, Iran; 2 Department of Community Medicine, Babol University of Medical Sciences, Babol, Mazandaran, Iran; 3 Department of Psychiatry, Babol University of Medical Sciences, Babol, Mazandaran, Iran; 4 Department of Psychiatry, Shahid Beheshti University of Medical Sciences, Tehran, Iran; Qazvin University of Medical Sciences, ISLAMIC REPUBLIC OF IRAN

## Abstract

Epilepsy, particularly in older people, is associated with significant changes in physical activities, social and occupational functions, and diet. It is associated with significant changes in physical activities, social and occupational functions, and diet. Valid and reliable instruments are needed to assess these changes. This study aimed to develop and validate a questionnaire to assess lifestyle in older people with epilepsy, named the Epilepsy Lifestyle Questionnaire (ELQ). This methodological study was conducted in 2022. The primary ELQ items were generated through reviewing the literature. Then, its face, content, construct, convergent, and discriminant validity, internal consistency, and test-retest stability were evaluated. Older patients aged ≥ 60 years were recruited from the Iranian Epilepsy Association to assess construct validity using confirmatory factor analysis (CFA). Data were analyzed using the SPSS (v. 26.0), AMOS (v. 24.0), and JASP software. The primary ELQ had 99 items with five subscales, namely health-oriented self-care, risk-averse behavior, emotional and psychosocial adaptation, epilepsy stigma, and intimacy and sexual behaviors. Sixty five items were deleted during psychometric evaluation. CFA showed the good fitting of the five-factor structure of the 34-item ELEQ (PCFI = 0.741, PNFI = 0.693, CMIN/DF = 0.073, IFI = 0.917, CFI = 0.916, AND GFI = 0.902). The values of internal consistency based on Cronbach’s alpha and test-retest reliability based on intraclass correlation coefficient (ICC) of ELQ were 0.89 and 0.95, respectively. The ELQ is a valid and reliable self-report instrument. The results suggest that the ELQ is a useful clinical tool for assessing the lifestyle of patients with epilepsy.

## Introduction

Epilepsy is a chronic, noncommunicable disease of the brain. Approximately, 50 million people worldwide suffer from epilepsy. Recurrent partial or generalized seizures, and sometimes loss of consciousness and bowel or bladder control are among the consequences of this disease [1].

The prevalence of epilepsy is 4–10 case per 1000 people in the world and eighteen cases per 1000 people in Iran [2]. The incidence and the prevalence of epilepsy among elderly people are higher than the youth [3]. The annual incidence of status epilepticus is 15.5 cases per 100000 people aged 60–69 years, 21.5 cases per 100000 people aged 70–79 years, and 24.9 cases per 100000 people aged eighty years and more [4]. The prevalence of epilepsy in Iranian older adults was 35 from 1482 participants (24/1000) [5]. Living with epilepsy necessitates significant changes in lifestyle [6]. Lifestyle refers to a set of activities, interests, and beliefs [7] that significantly affect health [8]. A healthy lifestyle consists of a healthy diet, regular physical and recreational activities, avoidance of tobacco, alcohol, and addictive substances, and good social relationships [9]. The lifestyle of chronically ill patients also includes disease-related aspects [10], because living with a chronic disease results in significant lifestyle changes [11]. Patients with chronic diseases need to change their diet and physical activity and adhere to treatment. Ineffective coping with disease-related lifestyle changes can make treatment difficult [12]. Adult patients with epilepsy (PWE) need to undergo positive changes in lifestyle such as alcohol abstinence, smoking cessation, avoidance of competitive exercises, and having a regular sleep pattern. Almost 50% of PWE report the negative effects of epilepsy on their lives. These lifestyle changes are especially necessary for patients with uncontrolled epilepsy [13].

The results of the study by Tedrus (2017) indicated that the most PWE are sedentary and do not practice physical activity (PA) for fear of seizures [14]. Sleeth (2016) reported that 70 percent of the participants in study having experienced felt stigma [15]. Adults with epilepsy smoked more and did less physical activity than adults without epilepsy [16]. The prevalence of overweight and obesity in adults with epilepsy is 66.7%, which indicates an unhealthy lifestyle [17].

Escoffery (2016) assessed self-management behaviors among adults with epilepsy and explored differences in behaviors among different age groups (18–29, 30–49, and 50). The results indicated the effect of epilepsy on dimensions such as bothered by seizures (F (2, 414) = 7.23, p = .001), being bothered by work limitations (F (2, 414) = 3.78, p = .024), and being bothered by the mental effects of antiepileptic medicine. Younger adults with epilepsy (<30 years) reported fewer safety, health and treatment behaviors than other adults. In general, all samples with different age groups performed less life style behaviors. Perception of different age groups in self-management and life style behaviors among adults with epilepsy can help healthcare system interventions to provide training for self-care promotion and sharing of medical decisions [18].

For effective coping with epilepsy and positive lifestyle changes, patients require strong support from families, healthcare providers, and society [19]. Nurses, as the largest group of healthcare providers, need to support positive lifestyle changes in PWE [20]. Any attempt to make positive lifestyle change in PWE requires careful assessment of lifestyle and lifestyle behaviors through valid and reliable instruments [21] appropriate to the cultural context of the society [22].

Previous studies have assessed lifestyle in PWE using instruments that were not specifically designed to assess lifestyle in epilepsy [23–27] such as the Health Lifestyle Assessment Questionnaire in the Elderly [28], the Health-Promoting Lifestyle Profile [29], the Lifestyle Personality Inventory [30], the Healthy Lifestyle and Personal Control Questionnaire [31], the Health and Lifestyle Questionnaire [32], the Healthy Lifestyle Questionnaire [33], the Self-Management of Epilepsy Questionnaire [34], the Quality of Life in Epilepsy Questionnaire [35], the Epilepsy Self-Efficacy Scale [36], the Epilepsy Risk Awareness Scale [37], the Epilepsy Knowledge Scale [38], the Life Habits Questionnaire [39], the Social and Occupational Functioning Scale for Epilepsy [40], the Stigma Scale of Epilepsy [41], and the Epidaily Scale [42].

All available instruments measure general lifestyle or concepts such as disease management, quality of life, knowledge, stigma, and self-efficacy in PWE. However, the construct of epilepsy-related lifestyle differs from these concepts. Epilepsy and the management of its symptoms such as seizures and the limitations it imposes on the patient’s personal and social life result in significant changes in all aspects of lifestyle such as adherence to therapeutic diet, sleep, exercise and leisure, social stigma, sexual behavior, tobacco and alcohol use. Therefore, there is a need for an instrument capable of measuring the lifestyle changes in PWE in all of the aforementioned aspects. Healthcare providers need a tool to detect unhealthy lifestyles and plan and implement evidence-based measures during follow-up for these patients. The lack of a specific instrument for lifestyle assessment in PWE highlights the need for studies to develop a valid and reliable instrument in this area. The present study sought to narrow this gap by developing and validating a questionnaire to assess the lifestyle of older people with epilepsy, named the Epilepsy Lifestyle Questionnaire (ELQ).

## Materials and methods

### Type of study

This methodological study was conducted in two main phases in 2022: the ELQ development and the ELQ psychometric evaluation.

### Phase one: The ELQ development

The primary item pool of the ELQ was developed by reviewing the literature. Accordingly, the PubMed, ScienceDirect, Google Scholar, Scopus, and Persian databases were searched using the keywords “older adulthood,” “lifestyle,” “self-management,” “self-efficacy,” “quality of life,” “disease-related quality of life,” “treatment adherence,” “coping with epilepsy,” “nutrition among PWE,” “physical activity among PWE,” “instrument,” “scale,” and “questionnaire” in publications until 2020. Search results included quantitative and qualitative studies on PWE experiences of stigma [43], physical activity among PWE [15], development of an epilepsy self-management instrument for adults [24], development of a lifestyle questionnaire for older adults [44], effects of lifestyle modification on PWE outcomes [45], do people with epilepsy have a different lifestyle? [46], lifestyle, disease acceptance, and lifestyle modification among PWE [13]. Peer-reviewed articles published in English and Persian languages were included. The exclusion criteria were editorial and commentarial materials and articles not available in full text. In the initial search, 48 studies (36 English and 12 Persian) were obtained. After excluding duplicate and irrelevant studies, 29 studies (24 English and five Persian) were included and subjected to content analysis to prepare the initial item pool.

### Phase two: The ELQ psychometric evaluation

In this phase, the face, content, and construct validity as well as the reliability of ELQ were evaluated.

### Face validity assessment

Face validity was evaluated using qualitative and quantitative techniques. In the qualitative assessment of face validity, ten older people were invited to comment on the difficulty, appropriateness, and clarity of the items [47]. In the quantitative assessment of face validity, ten experts in nursing, neurology, and psychology who were experienced in instrument development or providing care to older PWE were asked to rate the items on a five-point scale from “Not important” to “Completely important”. Items with an impact score of 1.5 or higher were kept and other items were excluded. The impact score was calculated through the “Frequency (%) × Importance” formula [47].

### Content validity assessment

Content validity was also assessed using qualitative and quantitative techniques. In the qualitative assessment of content validity, ten experts with knowledge and experience in the field of epilepsy-related lifestyle were asked to provide written comments on the wording, grammar, allocation, and scoring of the items [48]. In the quantitative assessment of content validity, the content validity ratio (CVR) and content validity index (CVI) were calculated.

CVR calculation: Initially, ten experts in epilepsy-related lifestyle were asked to rate the essentiality of each item as “Essential,” “Useful but not essential,” or “Inessential” [47]. According to Lawshe, the minimum acceptable CVR is 0.62 for ten experts [49]. The CVR was calculated using the CVR strict method [50], in which only essential items were included. The CVR was calculated through the “CVR = (ne − N/2)/ (N/2)” formula, where N was the total number of experts, and ne was the total number of experts who rated the intended item “Essential.”

*CVI calculation*: The same ten experts were asked to rate the relevance of the items on a four-point scale from “Irrelevant” to “Completely relevant”, and items with a CVI value greater than 0.79 were considered appropriate [51]. The CVI was calculated using the following formula [50]:

$$CVI=\frac{Number\;of\;raters\;who\;rated\;the\;item\;3\;or\;4}{Total\;number\;of\;the\;raters}$$

### Construct validity evaluation

The construct validity of the ELQ was evaluated by confirmatory factor analysis (CFA) because the factor structure of the primary ELQ had already been determined through the textual content analysis of the literature review data. Moreover, there was limited number of eligible older PWE in the study setting to be included in exploratory factor analysis. Model fit in CFA was evaluated using the following indices: Chi-square divided by degree of freedom (CMIN/DF), parsimonious normed fit index (PNFI), comparative fit index (CFI), parsimonious comparative fit index (PCFI), incremental fit index (IFI), goodness of fit index (GFI), and root mean square error of approximation (RMSEA). The necessary data for the CFA were collected in a cross-sectional study.

### Participants

Participants were purposefully selected from the Iranian Epilepsy Association, Tehran, Iran. Inclusion criteria were age 60 years and older, married, basic literacy skills, a definite diagnosis of epilepsy at least one year before enrollment in the study, access to the Internet, WhatsApp and Telegram, no hearing or visual impairment affecting the ability to establish interpersonal communication and no known psychological disorders.

Individuals who withdrew from the study or were reluctant to answer the questionnaire were excluded from the study. There is no formula or calculation to estimate the sample size for CFA, however, according to researchers such as Kline (2023), at least 200 individuals are required for a sufficient sample size [52]. In this study, the sample size was increased to 210 due to a probable attrition rate of 5%. To measure the test-retest reliability, we used 30 samples who completed the instrument in the first two phases and three weeks later. To account for attrition in sampling, we added 15% to the original sample size [52].

### Data collection

Data collection instruments were designed using Porsline software, and the link was sent to participants via WhatsApp and Telegram. Online data collection was chosen due to the significant decrease in patients’ attendance at healthcare settings or physicians’ offices during the COVID-19 pandemic. We initially referred to the study setting and created a list of eligible PWE and their phone numbers. The number of older people of Epilepsy Society was 507. The files available in the Epilepsy Society were used to check some of the inclusion criteria. Some other inclusion criteria were also checked through phone calls to the participants. Before sending the instruments, the study objectives were explained to the participants and their initial consent was taken. Before distributing the data collection tool, an informed consent form was sent to the participants via WhatsApp and Telegram. Upon completion and signature by the participants, the study tool was then sent to them. Participants were asked to complete the instruments themselves. Then, the research instruments were sent to them.

### Evaluation of data distribution, outliers, and missing data

Data normality and outliers were evaluated through both univariate and multivariate distribution evaluations. Multivariate outliers were evaluated using Mahalanobis distance (P < 0.001), while multivariate kurtosis was evaluated using Mardia’s coefficient (more than 20) [53]. Missing values were also assessed through multiple imputations and were replaced with the mean scores of participants.

### Convergent and discriminant validity assessment

After construct validity evaluation, convergent and discriminant validity were assessed using Fornell and Larcker’s indices, composite reliability (CR), average variance extracted (AVE), maximum shared squared variance (MSV), and average shared squared variance (ASV). Convergent validity refers to strong correlations among the items of a factor, whereas discriminant validity refers to the independence of the extracted factors from each other. Convergent validity is confirmed when the AVE exceeds 0.5, the CR exceeds 0.7 and is more than the AVE, while discriminant validity is confirmed when the MSV and ASV are less than the AVE [54].

### Test-retest reliability

Intra-class Correlation Coefficient (ICC) was used to measure the test-retest reliability of the ELQ. The absolute agreement method with a two-way random model was used to check the ICC. The test-retest method assumes that the variables, concepts, and characteristics of the participants will not change. The findings were interpreted as follows: slight (0–0.20), fair (0.21–0.40), moderate (0.41–0.60), substantial (0.61–0.80), and almost perfect (0.81–1.0) [55].

### Standard error of measurement (SEM) evaluation

SEM was evaluated using the following formula, $SEM=SD\times \sqrt{1-ICC}$, where *SD* is the standard deviation and *ICC* is the intraclass correlation coefficient. Small SEM values are significant because changes larger than SEM are considered clinically significant [56]. Moreover, the agreement parameter of the instrument was determined through the minimum detectable change (MDC) and the minimally important change (MIC). The formulas for calculating MDC and MIC were $MDC=SEM\times Z\;score\times \sqrt{2}$ and MIC=0.5×SDofΔscore, respectively. The agreement parameter is positive when MDC is greater than MIC. In fact, MDC is an actual change, which is not due to measurement error [56].

### Ceiling and floor effects assessment

Ceiling and floor effects are calculated based on the number of respondents scoring the highest and lowest possible scores, respectively. They are present when more than 15% of respondents achieve the highest or lowest possible scores. These effects indicate the likely absence of items reflecting the maximum and minimum severity of the intended phenomenon in the instrument, the ability of the instrument to differentiate between respondents achieving the lowest and highest scores, and the unacceptable content validity and reliability of the instrument. Ceiling and floor effects can be measured by skewness. A positive skewness reflects the floor effect, while a negative skewness reflects the ceiling effect [57].

### Reliability

Internal consistency was evaluated using Cronbach’s alpha, theta coefficient, McDonald omega coefficient, and CR. In this study, values higher than 0.7 were considered acceptable [58].

### Statistical analyses

SPSS software v26.0 (SPSS Inc., Chicago, IL) was used to describe the data and calculate the test-retest ICC. CFA was performed using AMOS software v24.0 (IBM SPSS Amos., United States), and the McDonald omega coefficient was calculated using JASP software (Jeffreys’s Amazing Statistics Program., Amsterdam).

### Ethics statement

All methods were carried out in accordance with relevant guidelines and regulations, such as the Declaration of Helsinki. The ethical approval for this study was obtained from the Ethics Committee of Babol University of Medical Sciences (Consent number: IR.MUBABOL.HRI.REC.1398.327). Written and verbal consent was obtained from all participants. We informed participants of the study aim, confidential data management, and their freedom to unilaterally withdraw from the study.

## Results

### Demographic and clinical information of participants

Participants were 210 older PWE with a mean age of 65.67±5.50 years. Almost half (50.5%) of the participants were female, and had lower/upper secondary education (50.5%) (Table 1).

**Table 1:** Participants’ demographic characteristics.

Characteristics		N	%
**Gender**	**Male**	104	49.5
	**Female**	106	50.5
**Occupation**	**Housewife**	58	27.6
	**Retired**	77	36.7
	**Employee**	20	9.5
	**Self-employed**	55	26.2
**Educational level**	**Below diploma**	106	50.5
	**Diploma**	48	22.9
	**Associate diploma**	24	11.4
	**Bachelor’s and higher**	32	15.2
**Income**	**Sufficient**	57	27.1
	**Relatively sufficient**	89	42.4
	**Insufficient**	64	30.5
**Insurance coverage**	**No**	48	22.9
	**Yes**	162	77.1
**Additional insurance coverage**	**No**	105	50
	**Yes**	105	50
**Place of residence**	**Urban areas**	198	94.3
	**Rural areas**	12	5.7
**Living arrangement**	**With children**	31	14.8
	**With spouse and children**	101	48.1
	**With spouse**	28	18.1
	**Alone**	40	19
**History of tobacco use**	**No**	162	77.1
	**Yes**	48	22.9
**History of drug abuse**	**No**	205	97.6
	**Yes**	5	2.4
**Age (Years), Mean±SD**		65.67±5.50	

The mean age at onset of epilepsy was 22.88±18.21 years, and the mean duration of epilepsy was 33.81±19.56 years. Participants had received antiepileptic medications for a mean of 32.90±19.23 years (Table 2).

**Table 2:** Participants’ clinical characteristics.

Characteristics		N	%
**Number of seizures per month**	0	101	48.1
	1	23	11
	2	37	17.6
	3	19	9
	4	16	7.6
	> 4	14	6.7
**Disease status**	Uncontrolled	41	19.5
	Poorly controlled	64	30.5
	Controlled	105	50
**Family history of epilepsy**	No	132	62.9
	Yes	78	37.1
**Antiepileptic medications**	Carbamazepine	83	39.5
	Primidone	31	14.8
	Phenytoin	52	24.8
	Phenobarbital	42	20
	Valproat	45	21.4
	Levetiracetam	8	3.8
	Lamotrigine	2	1
	No medication	10	4.8
	Other medications	90	42.9
**Antiepileptic regimen**	Single medication	62	29.5
	Multiple medications	148	70.5
**Epilepsy type**	Primary	132	62.9
	Secondary	78	37.1
**Comorbid conditions**	Diabetes mellitus	27	12.9
	Cardiovascular disease	42	20
	Hypertension	28	13.3
	Cerebrovascular disorders	38	18.1
	Neurological disorders	22	10.5
	Orthopedic problems	42	20
	Hematological problems	16	7.6
	Immune deficiency	30	14.3
	Digestive disorders	48	22.9
	Hepatic problems	13	6.2
	Other problems	12	5.7
**Intake of non-antiepileptic medications**	No	66	31.4
	Yes	144	68.6
**Type of non-antiepileptic medications**	Cardiac	57	27.1
	Anti-diabetic	19	9
	Antihypertensive	21	10
	Vitamins	27	12.7
		
	Medications for digestive problems	20	9.5
**Age of affliction by epilepsy (Years), Mean ±SD**		22.88±18.21	
**Duration of affliction by epilepsy (Years), Mean ±SD**		33.81±19.56	
**Duration of using antiepileptic medications (Years), Mean ±SD**		32.90±19.23	

### Phase one: The ELQ development

At the end of phase one, 105 initial codes were extracted and categorized into five themes. The research team carefully studied the codes in regular meetings and converted them into items, creating an initial pool of 105 items. Duplicate, overlapping, and similar items were reviewed, and some items were merged or deleted. Therefore, the total number of items was reduced first to 99 and then to 64. Finally, a preliminary form of the ELQ was developed with five-point Likert response options (1 = never, 2 = rarely, 3 = sometimes, 4 = often, 5 = always).

### Phase two: The ELQ psychometric evaluation

**Face validity evaluation.** To qualitatively measure the face validity from the viewpoint of the target group, we modified the wording and appearance of a few items for better understanding. In the quantitative assessment of face validity, we removed seven items with scores lower than 1.5: “I feel hopeless because of my illness and old age,” “I monitor the side effects of anticonvulsants," “When one of my medications runs out, I refill it by taking another medication,” “When I leave the house, I take the medications with me,” “If I miss the medication dose, I follow my doctor’s instructions,” "I do not allow others treat me like a child," and “I ask family members and friends to help me with daily activities.” The ELQ included 57 items at the end of this step.

### Content validity evaluation

In the qualitative assessment of content validity of the 57-item ELQ, we combined two items and revised some items. The ELQ had 55 items at the end of this step. In the quantitative assessment of content validity, we deleted three items (“When my anticonvulsant runs low, I take less or use a similar medication,” “I do not abruptly stop my anticonvulsant medication,” and "I control my diet to avoid repeated seizures”) due to CVR values less than 0.62. The CVI values of all remaining 52 items were greater than 0.79.

### Construct validity evaluation

We evaluated the five-factor structure of the ELQ using the first-order factor analysis model. The five factors of this model were health-oriented self-care, risk-averse behavior, emotional and psychosocial adaptation, epilepsy stigma, and intimacy and sexual behaviors. The proposed model did not fit the data well. In the next step, we deleted eighteen items with factor loading values less than 0.3 to improve model fit, and then evaluated the correlation between measurement errors as well as the fit indices of the model before and after modification. The after modification Chi-square test value of the first model was 1036.71 with a DF of 484, and the test result was significant (P < 0.001) (Table 3).

**Table 3:** Model fit indices in confirmatory factor analysis.

Indices Model	χ^2^	df	P value	CMIN/df	RMSEA (90% CI)	PNFI	CFI	PCFI	IFI	GFI
**First order model before modification**	4129.32	1264	< 0.001	3.26	0.104(0.09–0.12)	0.520	0.861	0.549	0.867	0.834
**First order model after modification**	1036.71	484	< 0.001	2.14	0.073(0.07–0.08)	0.693	0.916	0.741	0.917	0.902
**Second order**	1061.15	489	< 0.001	2.17	0.074(0.07–0.08)	0.691	0.915	0.739	0.916	0.902

Abbreviations: CMIN/DF: Chi-square divided by degree of freedom; PNFI: Parsimonious normed fit index; CFI: Comparative fit index; PCFI: Parsimonious comparative fit index; IFI: Incremental fit index; GFI: Goodness of fit index; RMSEA: Root mean square error of approximation.

**Fig 1:**
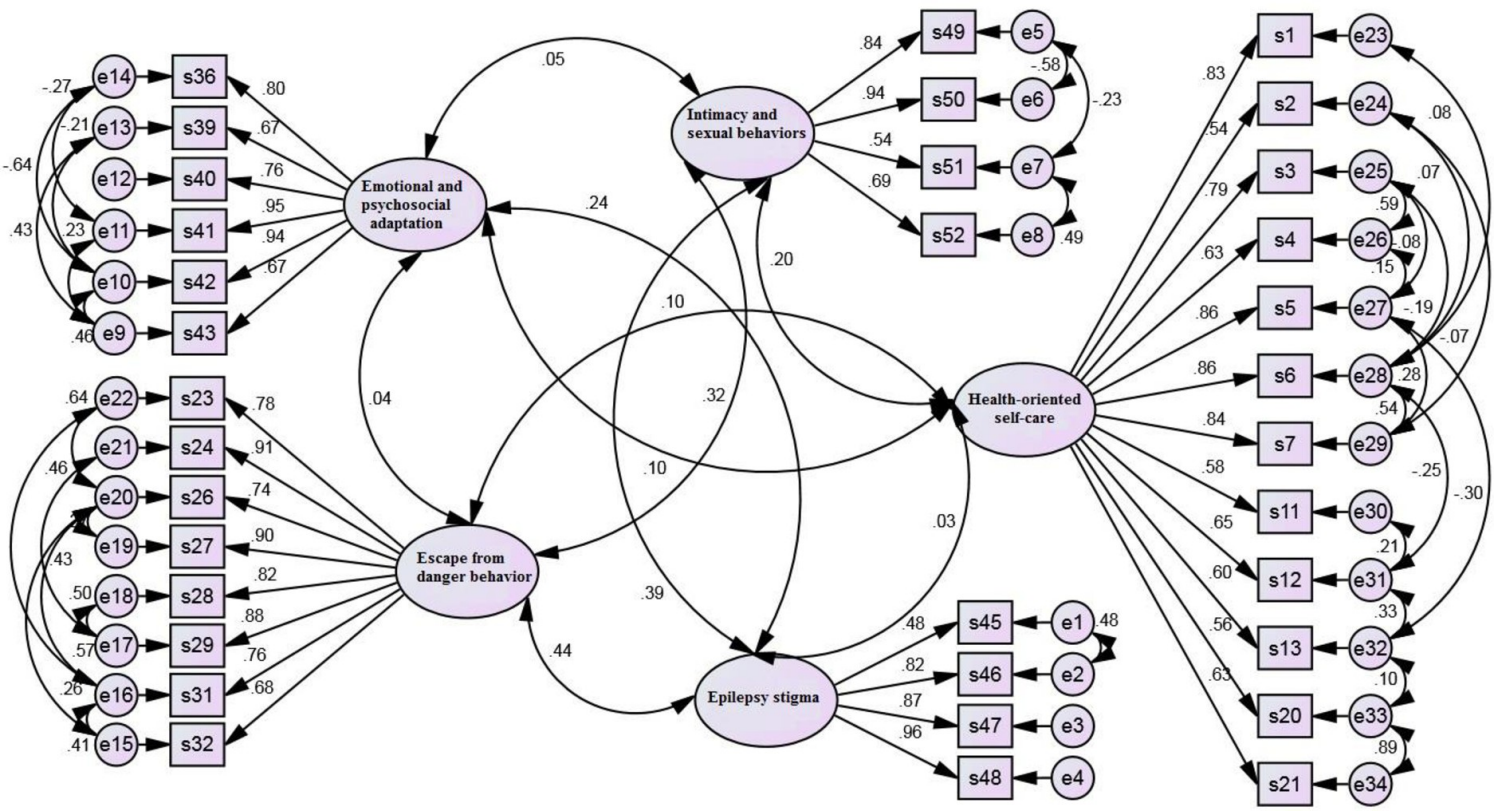
The first order confirmatory factor analysis model after modification.

In the corrected first-order factor analysis model, the standardized factor loading values of all remaining items were greater than 0.4 (Table 4 and Fig 1) and the Cronbach’s alpha of all factors after deleting each item was higher than 0.7.

**Table 4:** The standardized factor loading values of the ELEQ items.

No.	Item	Factor loading	Cronbach’s alpha after item removal	Factor Mean ±SD
1	I refer to my physician at the proper time for periodical examinations, laboratory tests, and electroencephalography.	0.827	0.911	Health-oriented self-care 44.98±8.53
2	I ask my physician about epilepsy treatment methods such as medication therapy, non-pharmacological therapies, and dietary regimen.	0.544	0.926	
3	I procure my anti-epileptic medications before they are finished.	0.794	0.912	
4	I take my anti-epileptic medications completely and on time.	0.631	0.917	
5	I inform my physician if my anti-epileptic medications cause me physical, mental, or memory-related problems.	0.861	0.912	
6	I refer to my physician if my seizure frequency increases.	0.864	0.912	
7	I inform my physician in case of change in seizure type (from local to generalized).	0.843	0.912	
11	I prevent weight gain" vs. being mindful of weight gain.	0.583	0.919	
12	I provide my significant others with education about appropriate measures during seizure.	0.645	0.915	
13	I ask help from other patients with epilepsy in order to cope with my disease.	0.597	0.919	
20	I regularly perform physical exercise.	0.564	0.917	
21	I walk at least thirty minutes per day.	0.630	0.914	
23	In the bathroom, I use shower instead of the tub.	0.775	0.942	Escape from danger behavior 32.6±5.03
24	I ask my significant others to stay home when I want to perform high-risk activities such as bathing.	0.908	0.938	
26	I control water temperature at home or in the bathroom to prevent burn injury in case of seizure.	0.740	0.941	
27	When I’m alone at home, I get ensured that the gas valve is closed and cigarette or candle is extinguished.	0.895	0.939	
28	I sit or lie if I feel that I may experience seizure.	0.823	0.942	
29	I observe safety precautions such as leaving bathroom door open, covering furniture edges, and using thick floor coverings at home.	0.877	0.937	
31	I identify and avoid factors which can cause seizure such as severe stress, worry, fatigue, and sleeplessness.	0.763	0.940	
32	I use safety helmet and other personal protective equipment during physical exercise or going anywhere with fall risk such as high altitudes.	0.677	0.950	
33	I communicate with individuals who know about epilepsy.	0.798	0.907	Emotional and psychosocial adaptation 21.39±6.59
39	I don’t attempt to fulfill my wishes or pursue my goals because I don’t see a clear future with my disease.	0.671	0.910	
40	I get angry at others’ compassionate reactions towards my disease.	0.764	0.905	
41	It makes me unhappy that others keep aloof from me due to my disease.	0.950	0.988	
42	I get angry over others’ fear of me due to my epilepsy.	0.940	0.886	
43	I ask emotional support from my family, friends and caregivers.	0.674	0.899	
45	I don’t talk about my disease with those who don’t know about epilepsy.	0.476	0.918	Epilepsy stigma 10.50±5.32
46	I avoid social activities in order not to make my family ashamed.	0.823	0.789	
47	I avoid going out of home due to the probability of seizure.	0.874	0.829	
48	I avoid group sport or recreational activities so that others do not notice my disease.	0.964	0.805	
49	I have no interest in sexual relationship since affliction by epilepsy.	0.838	0.837	Intimacy and sexual behaviors 9.84±4.74
50	The notion that sexual behavior can lead to seizure has made me avoid sexual relationship with my spouse/partner.	0.942	0.746	
51	The notion that my spouse/partner may reject my request for sexual behavior due to my disease makes me avoid sexual relationship.	0.544	0.834	
52	Fear over seizure during sexual relationship has made me avoid sexual activity.	0.689	0.755	

**Fig 2:**
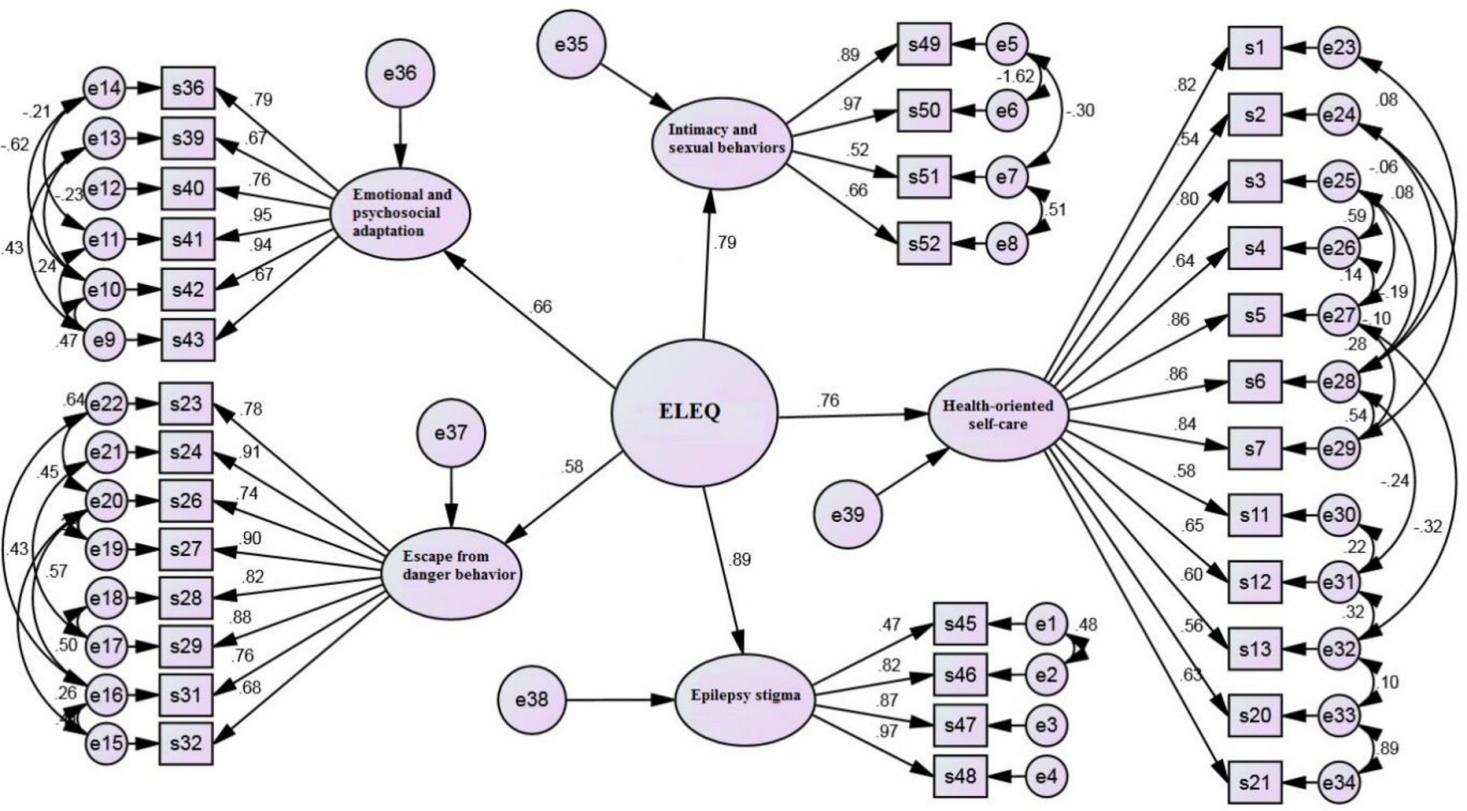
The second confirmatory factor analysis model.

Then, we used CFA for the second time to determine the relationship of the five factors with the construct of epilepsy-related lifestyle and their contribution to explaining the variance of the construct. The results showed that all model fit indices were acceptable, confirming the goodness of model fit (Table 3 and Fig 2).

### Convergent and discriminant validity assessment

In the first CFA, the AVE values of all factors were greater than 0.5 (0.503–0.658), and the AVE value of each factor was also greater than the values of its ASV (0.014–0.081) and MSV (0.040–0.193) (Table 5).

**Table 5:** The results of the evaluation of convergent and discriminant validity, internal consistency, and stability of ELEQ.

No.	Parameters Factors	*α*	*θ*	Ω	ICC (90% CI)	First model				Second model	
						CR	ASV	MSV	AVE	CR	AVE
1	**Health-oriented self-care**	0.922	0.920	0.918	0.91 (0.82–0.96)	0.922	0.006	0.040	0.503	0.858	0.553
2	**Escape from danger behaviors**	0.948	0.941	0.940	0.88 (0.76–0.94)	0.938	0.061	0.193	0.658		
3	**Emotional and psychosocial adaptation**	0.914	0.910	0.907	0.83 (0.66–0.92)	0.917	0.014	0.057	0.652		
4	**Epilepsy stigma**	0.873	0.866	0.863	0.85 (0.69–0.93)	0.875	0.081	0.193	0.649		
5	**Intimacy and sexual behaviors**	0.838	0.832	0.830	0.92 (0.85–0.96)	0.847	0.059	0.152	0.590		
6	**Total**	0.897	0.895	0.893	0.95 (0.90–0.97)	—	—	—	—	—	—

Abbreviations: α: Cronbach’s alpha; θ: theta coefficient; Ω: McDonald omega; CR: Composite reliability; AVE: Average variance extracted; MSV: Maximum shared squared variance; ASV: Average shared squared variance; ICC: Intraclass correlation coefficient.

Moreover, there were weak to moderate pairwise correlations between the factors of ELQ, denoting their actual discrimination from each other (Table 6). These results confirm the acceptable convergent and discriminant validity of the ELQ.

**Table 6:** The coefficients of pairwise correlations among the ELEQ factors.

No.	Factors	1	2	3	4
1	Health-oriented self-care	1			
2	Escape from danger behaviors	0.091	1		
3	Emotional and psychosocial adaptation	^a^0.214	^a^0.186	1	
4	Epilepsy stigma	0.028	^a^0.331	^a^0.498	1
5	Intimacy and sexual behaviors	^a^0.314	^a^0.338	0.044	^a^0.355

a; P<0.01

### SEM evaluation

As the agreement parameter of the ELQ was positive, the MDC was actual and not due to measurement error (Table 7). The results suggest that the use of the ELQ at different times has the lowest measurement error and can show the lowest changes in clinical studies.

**Table 7:** The standard error of ELEQ.

No.	Factors	Mean±SD	Range	SEM	MDC	MIC	Agreement
1	**Health-oriented self-care**	44.98±8.53	16–60	2.559	7.09	0.49	Positive
2	**Escape from danger behaviors**	32.36±5.03	19–40	1.742	4.82	0.375	Positive
3	**Emotional and psychosocial adaptation**	21.39±6.59	6–30	2.717	7.53	0.42	Positive
4	**Epilepsy stigma**	10.50±5.32	4–20	2.060	5.71	0.405	Positive
*5*	**Intimacy and sexual behaviors**	9.84±4.74	0–20	1.340	3.71	0.28	Positive
	**Total**	119.08±17.28	72–146	3.863	10.71	1.28	Positive

### Ceiling and floor effects assessment

The participants who scored the lowest and the highest ELQ scores were both less than 15%. Therefore, the ELQ had no ceiling and floor effects and its scores had normal distribution (Table 8). The results indicate that the ELQ does not consist of the items with the maximum and minimum intensity, so patients with the minimum and maximum scores are differentiated and its reliability also increased.

**Table 8:** The ceiling and the floor effects of ELEQ.

No.	Factors	Ceiling effect	Floor effect	Skewness	Kurtosis
1	**Health-oriented self-care**	4%	0%	–0.614	0.463
2	**Escape from danger behaviors**	7.6%	0%	-0.327	-0.687
3	**Emotional and psychosocial adaptation**	8.6%	4.3%	-0.900	-0.025
4	**Epilepsy stigma**	9.5%	7.5%	-0.430	-1.571
5	**Intimacy and sexual behaviors**	6.7%	8.4%	-0.526	-0.611
	**Total**	5%	0%	-0.516	0.283

### Instrument scoring

The 34 items of the ELQ are scored on a five-point scale as follows, 1: “Never”; 2: “Rarely”; 3: “Sometimes”; 4: “Often”; and 5: “Always”, with higher scores indicating higher levels of life style and lower scores indicating lower levels of life style related to epilepsy. The ELQ score was obtained from the total number of items. For the leveling of the total ELQ score, the equal distance method has been used. In this way, we divided the distance between 34 and 170 into five equal parts. The total score of the questionnaire varies from 34 to 170: scores 34–61, 62–88, 89–115, 116, 142, and 143–170 indicate very poor, poor, moderate, good, and very good lifestyle, respectively.

### Reliability

The results revealed that Cronbach’s alpha, Macdonald’s omega and CR of all factors were higher than 0.7, and internal consistency reliability value for all factors was higher than 0.8. These findings show acceptable and appropriate internal consistency reliability or reproducibility of epilepsy-related lifestyle over time. Therefore, the reliability of the ELQ is at a favorable and acceptable level (Table 5).

## Discussion

The purpose of this study was to develop and evaluate the psychometric properties of the ELQ as a screening tool for PWE. This is the first study that designs a tool and reports its validity and reliability indicators in Iranian older adults with epilepsy. The ELQ had acceptable validity and reliability. The final ELQ included 34 items in five subscales, namely health-oriented self-care, risk-averse behavior, emotional and psychosocial adaptation, epilepsy stigma, and intimacy and sexual behaviors. Items were scored on a five-point Likert scale. Likert scales are three-, four-, six-, or seven-point and scores of instruments with Likert scale are treated at interval measurement level [59].

Content validity evaluation based on the comments of ten experts revealed that the 34 items of the ELQ had acceptable CVR and CVI values. Content validity assesses whether the items of the intended instrument are appropriate for measuring the intended phenomenon [49]. Evidence shows that nine experts are adequate to assess content validity [60].

The construct validity of the ELQ was evaluated using CFA to assess the latent factor structure of the instrument, confirm the number of factors, and identify the pattern of item-factor relationships [61]. In the present study, PCFI, PNFI, CMIN/DF, RMSEA, IFI, CFI, and GFI model fit indices confirmed the good fit of the confirmatory factor model of the ELQ. Moreover, the factor loading values of all items were above 0.4 after model correction [62]. After removal of each item, the Cronbach’s alpha values of the ELQ factors were still greater than 0.7 [62]. Moreover, there were weak to moderate pairwise correlations between the ELQ factors, confirming that the factors are discriminant from each other. Discriminant validity denotes that the extracted factors are independent of each other [63]. The questionnaire had acceptable convergent validity, denoting that its items are close together and share great variance with each other.

The theta coefficient, McDonald omega, Cronbach’s alpha, and test-retest ICC values of the ELQ and all its factors were greater than 0.8, confirming the acceptable reliability of the questionnaire [62]. Internal consistency shows that the different items of an instrument measure a same construct [64]. When evaluating internal consistency, the Cronbach’s alpha value of an item should be at least 0.7 to keep that item in the instrument [65]. The test-retest stability assessment method assumes that the intended instrument and respondents’ characteristics do not change significantly over time. The ICC is the most acceptable statistical method for assessing stability [66]. ICC values greater than 0.8 confirm good stability, values of 0.6–0.8 confirm acceptable stability, and values less than 0.6 confirm low stability [67]. Stability is a substitute for Cronbach’s alpha in structural equation modeling [58]. Moreover, the results of SEM evaluation in the present study revealed that the ELQ has acceptable measurement precision and reliability. There are some levels of difference and change in all measurements due to the existence of some levels of measurement error. This was the first study of its kind that assessed lifestyle among older PWE in Iran. However, the ELQ can be used to examine epilepsy-related lifestyle and to identify and manage unhealthy lifestyle behaviors among PWE in other contexts. Among the study limitations were online data collection through self-report and development of the ELQ through a deductive method based on existing literature. Moreover, participants were older PWE who had access to the internet and might have not accurately represented all older PWE.

## Conclusion

The 34-item ELQ has acceptable validity and reliability. Its five subscales are health-oriented self-care, risk-averse behavior, emotional and psychosocial adaptation, epilepsy stigma, and intimacy and sexual behaviors. Items are scored on a five-point scale and the total possible score ranges from 34 to 170. Higher ELQ scores show better lifestyle in epilepsy. The ELQ is a self-report instrument and can be used for all older PWE who can communicate verbally and are oriented to time and place. Although the target group of the current study was older people, the ELQ is not specific to older people and can also be used for adults with epilepsy. Diagnosing unhealthy cases in different dimensions of lifestyle in patient follow-up by doctors and nurses in clinics, offices and hospitals can lead to evidence-based interventions. This instrument can also be used by caregivers to screen the lifestyle of PWE due to its simplicity, small number of items, and clear scoring.

## Supporting information

S1 Data (CSV)

S2 Data (XLSX)

## Abbreviations

ELQ: Epilepsy Lifestyle Questionnaire
CVR: Content validity ratio
CVI: Content validity index
ICC: Intraclass correlation coefficient
PAF: Principal axis factoring
CMIN/DF: Chi-square degree-of-freedom ratio
χ 2: Chi-square
AGFI: Adjusted Goodness-of-Fit Index
PCFI: Parsimonious Comparative Fit Index
CFI: Comparative Fit Index
IFI: Incremental Fit Index
PNFI: Parsimonious Normed Fit Index
RMSEA: Root Mean Square Error of Approximation
AVE: Average Variance Extracted
MSV: Maximum Shared Squared Variance
ASV: Average Shared Squared Variance

## References

[1] Wold Health Organization. Epilepsy. Available at: https://www.who.int/news-room/fact-sheets/detail/epilepsy

[2] Lin CY, Updegraff JA, Pakpour AH. The relationship between the theory of planned behavior and medication adherence in patients with epilepsy. Epilepsy Behav. 2016; 61: 231–236. doi: 10.1016/j.yebeh.2016.05.030 27390026

[3] Leppik I.E., Walczak T.S., and Birnbaum A.K.J.T.L., Challenges of epilepsy in elderly people. The Lancet. 2012; 380:1128–1130. doi: 10.1016/S0140-6736(12)61517-7 23021270

[4] Malter MP, Nass RD, Kaluschke T, Fink GR, Burghaus L, Dohmen C. New onset status epilepticus in older patients: Clinical characteristics and outcome. Seizure. 2017; 51:114–120. doi: 10.1016/j.seizure.2017.08.006 28843069

[5] Saadat P, Ahangar AA, Hosseini SR, Bijani A, Khalili M, Alijanpour S. Epilepsy and associated factors in elderly people of Amirkola, North of Iran (The Amirkola Health and Ageing Project). Caspian J Intern Med. 2023;14:100–107. doi: 10.22088/cjim.14.1.100 36741496 PMC9878904

[6] Szałwińska K., Cyuńczyk M., Kochanowicz J. and Witkowska A.M. Dietary and lifestyle behavior in adults with epilepsy needs improvement: a case-control study from northeastern Poland. Nutr J. 2021; 20: 62. doi: 10.1186/s12937-021-00704-6 34187474 PMC8243538

[7] Rahimi A, Anosheh M, Ahmadi F, Foroughan M. Exploring the nature of the elderly people life style: a grounded theory. Iranian J Age. 2016; 10:112–131.

[8] Gnardellis C, Tzamalouka G, Papadakaki M, Chliaoutakis JE. Aninvestigation of the effect of sleepiness, drowsy driving, and lifestyle on vehicle crashes. transportation research part F: traffic psychology and behaviour. 2008; 11: 270–281. doi: 10.1016/j.trf.2008.01.002

[9] Movahedi M, Khamseh F, Ebadi A, Haji Amin Z, Navidian A. Assessment of the lifestyle of the elderly in Tehran. J Health Promot Manage. 2016; 5: 51–59.

[10] Babak A, Davari S, Aghdak P, Pirhaji O. Assessment of healthy lifestyle among elderly in Isfahan, Iran. J Isfahan med school. 2011; 29: 1064–1074.

[11] Parsamehr M, Rasoulinezhad SP. The study of the relationship between lifestyle and social health among people of Talesh City. Quarterly Journal of Social Development. 2015; 10: 35–66.

[12] Shamsalinia A, Ghadimi R, Rad RE, Ghoozlu KJ, Mahmoudian A, Moradi M, Masoudi R, Ghaffari F. Psychometric Properties of the Persian Version of Adult Eating Behavior Questionnaire in Patients with Epilepsy. Iran J Med Sci. 2022; 47(3):236. doi: 10.30476/ijms.2021.89396.2011 35634526 PMC9126902

[13] Staniszewska A, Religioni U, Dąbrowska-Bender M. Acceptance of disease and lifestyle modification after diagnosis among young adults with epilepsy. Patient Prefer Adherence. 2017; 11: 165–174. doi: 10.2147/PPA.S126650 28203060 PMC5293500

[14] Tedrus GM, Sterca GS, Pereira RB. Physical activity, stigma, and quality of life in patients with epilepsy. Epilepsy Behav. 2017; 77: 96–98. doi: 10.1016/j.yebeh.2017.07.039 29033118

[15] Sleeth C, Drake K, Labiner DM, Chong J. Felt and enacted stigma in elderly persons with epilepsy: A qualitative approach. Epilepsy Behav. 2016; 55: 108–112. doi: 10.1016/j.yebeh.2015.12.026 26773679

[16] Cui W., Zack M.M., Kobau R. and Helmers S.L. Health behaviors among people with epilepsy—results from the 2010 National Health Interview Survey. Epilepsy Behav. 2015; 44: 121–126. doi: 10.1016/j.yebeh.2015.01.011 25678033 PMC4580240

[17] de Azevedo Fernandez R., Corrêa C., Bianchin M.M. and Perry I.D.S. Anthropometric profile and nutritional intake in patients with epilepsy. Nutr Hosp. (2015)32: 817–822. doi: 10.3305/nh.2015.32.2.9205 26268116

[18] Escoffery C., McGee R.E., Bamps Y. and Helmers S.L. Differences in epilepsy self-management behaviors among young and older adults. Austin J Neurol Disord Epilepsy. 2016; 3: 1015.

[19] Shamsalinia A, Masoudi R, Rad RE, Ghaffari F. Development and psychometric evaluation of the Perceived Social Stigma Questionnaire (PSSQ-for adults with epilepsy): A mixed method study. Epilepsy & Behavior. 2019; 96:141–9. doi: 10.1016/j.yebeh.2019.04.055 31146178

[20] Shamsalinia A, Moradi M, Farahani MA, Masoudi R, Ghadimi R, Rad RE, Ghaletaki GZ, Ghaffari F. Designing and psychometric evaluation of disease-related fear scale (D-RFS) in adults with epilepsy: A sequential exploratory mixed methods design. Epilepsy & Behavior. 2020; 110:107169. doi: 10.1016/j.yebeh.2020.107169 32504981

[21] Koivusilta L, Arja R, Andres V. Health behaviours and health in adolescence as predictors of educational level in adulthood: a follow-up study from Finland. Soc sci & med. 2003;57:577–93. doi: 10.1016/S0277-9536(02)00405-7 12821008

[22] Taghizadeh Z, Ebadi A, Montazeri A, Shahvari Z, Tavousi M, Bagherzadeh R. Psychometric properties of health related measures. Part 1: Translation, development, and content and face validity. Payesh (Health Monitor). 2017; 16:343–357.

[23] Saengsuwan J, Boonyaleepan S, Tiamkao S, Group IE. Diet, exercise, sleep, sexual activity, and perceived stress in people with epilepsy in NE Thailand. Epilepsy Behav. 2015; 45: 39–43. doi: 10.1016/j.yebeh.2015.02.014 25801753

[24] Capovilla G, Kaufman KR, Perucca E, Moshe SL, Arida RM. Epilepsy, seizures, physical exercise, and sports: a report from the ILAE Task Force on Sports and Epilepsy. Epilepsia. 2016; 57: 6–12. doi: 10.1111/epi.13261 26662920

[25] Ferrari CM, de Sousa RM, Castro LH. Factors associated with treatment non-adherence in patients with epilepsy in Brazil. Seizure. 2013; 22: 384–389. doi: 10.1016/j.seizure.2013.02.006 23478508

[26] Shi Y, Wang S, Ying J, Zhang M, Liu P, Zhang H, Sun J. Correlates of perceived stigma for people living with epilepsy: a meta-analysis. Epilepsy Behav. 2017; 70: 198–203. doi: 10.1016/j.yebeh.2017.02.022 28431368

[27] Tedrus GM, Pereira RB, Zoppi M. Epilepsy, stigma, and family. Epilepsy Behav. 2018; 78: 265–268. doi: 10.1016/j.yebeh.2017.08.007 29126703

[28] Bandari R, Mohammadi Shahboulaghi F, Montazeri A. Development and psychometric evaluation of the healthy lifestyle questionnaire for elderly (heal).Health and Quality of Life Outcomes. 2020; 18: 1–9. doi: 10.1186/s12955-020-01529-3 32787957 PMC7424645

[29] Tol A, Tavassoli E, Shariferad GR, Shojaeezadeh D. Health-promoting lifestyle and quality of life among undergraduate students at school of health, Isfahan university of medical sciences. Journal of education and health promotion. 2013; 28: 11. doi: 10.4103/2277-9531.108006 24083261 PMC3778574

[30] Wheeler M. S., & Acheson S. K. Criterion-related validity of the Life-Style Personality Inventory. Individual Psychology: Journal of Adlerian Theory, Research & Practice.1993; 49: 51–57.

[31] Darviri C, Alexopoulos EC, Artemiadis AK, Tigani X, Kraniotou C, Darvyri P, et al. The Healthy Lifestyle and Personal Control Questionnaire (HLPCQ): a novel tool for assessing self-empowerment through a constellation of daily activities. 2014; 14: 1–10. doi: 10.1186/1471-2458-14-995 25253039 PMC4192765

[32] Lali M, Abedi A, Kajbaf MB. Construction and validation of the lifestyle questionnaire (LSQ). 2012;15:64–80.

[33] Costa-Tutusaus L, Guerra-Balic M. Development and psychometric validation of a scoring questionnaire to assess healthy lifestyles among adolescents in Catalonia. BMC Public Health. 2015; 16: 1–12. doi: 10.1186/s12889-016-2778-6 26821644 PMC4731967

[34] Escoffery C, Bamps Y, LaFrance WC Jr, Stoll S, Shegog R, Buelow J, et al. Development of the adult epilepsy self-management measurement instrument (AESMMI).Epilepsy & Behav. 2015); 50: 172–183. doi: 10.1016/j.yebeh.2015.07.025 26303037

[35] Cramer JA, Perrine K, Devinsky O, Bryant‐Comstock L, Meador K, Hermann B. Development and cross‐cultural translations of a 31‐item quality of life in epilepsy inventory. Epilepsia. 1998;39: 81–88. doi: 10.1111/j.1528-1157.1998.tb01278.x 9578017

[36] DiIorio C, Yeager K. The epilepsy self-efficacy scale. Measurement of nursing outcomes: self care and coping. 2003; 3:40–51.

[37] Braun A, Kendall S, Cole C, Smeeton N, Angus-Leppan H. Development of the Epilepsy Risk Awareness scale (ERA scale) for people with epilepsy. Seizure. 2017;46: 13–18. doi: 10.1016/j.seizure.2017.02.005 28214711

[38] May TW, Pfäfflin M. The efficacy of an educational treatment program for patientswith epilepsy (MOSES): results of a controlled, randomized study. Epilepsia. 2002; 43: 539–549. doi: 10.1046/j.1528-1157.2002.23801.x 12027917

[39] Noreau L., Fougeyrollas P., and Vincent C. The LIFE-H: Assessment of the quality of social participation. Technology and disability.2002; 14: 113–118. doi: 10.3233/TAD-2002-14306

[40] Wang WH, Yu HY, Yen DJ, Lin YY, Kwan SY, Chen C, Hua MS. The Social and Occupational Functioning Scale for Epilepsy (SOFSE): A brief measure of functional status in a T aiwanese sample with epilepsy. Epilepsia. 2013;54:888–97. doi: 10.1111/epi.12141 23506167

[41] Fernandes PT, Salgado PC, Noronha AL, Sander JW, Li LM. Stigma scale of epilepsy: validation process. Arq Neuropsiquiatria. 2007; 65: 35–42. doi: 10.1590/s0004-282x2007001000006 17581666

[42] Gutiérrez-Viedma Á, Sanz-Graciani I, Romeral-Jiménez M, Parejo-Carbonell B, Serrano-García I, Cuadrado ML, Aledo-Serrano Á, Gil-Nagel A, Toledano R, Pérez-De-Heredia-Torres M, Santamarina E.Epidaily, a scale for comprehensive functional assessment of patients with epilepsy.Epilepsy & Behavior. 2021; 114: 107570. 10.1016/j.yebeh.2020.107570 33234457

[43] Forsgren L, Ghanean H, Jacobsson L, Richter J. On the experience of stigma by persons with epilepsy in Sweden and Iran—a comparative study. Seizure. 2013; 22: 748–751. doi: 10.1016/j.seizure.2013.05.016 23796412

[44] Eshaghi SR, Farajzadegan Z, Babak A. Healthy lifestyle assessment questionnaire in elderly: translation, reliability and validity. Payesh. 2010; 9: 91–99.

[45] Mohamed F, Abo Zead S, Shehata G, Abd-Almageed A. Effect of lifestyle modification on epileptic patients outcomes. Journal of Nursing and Health Science. 2018; 7:23–30. doi: 10.9790/1959-0704012330

[46] Aguirre C, Quintas S, Ruiz-Tornero AM, Alemán G, Gago-Veiga AB, de Toledo M, Vivancos J. Do people with epilepsy have a different lifestyle?. Epilepsy & Behav.2017; 74: 27–32. doi: 10.1016/j.yebeh.2017.06.006 28672217

[47] Bahariniya S, Ezatiasar M, Madadizadeh F. A Brief Review of the Types of Validity and Reliability of scales in Medical Research. Journal of Community Health Research. 2021;10:100–2. doi: 10.18502/jchr.v10i2.6582

[48] Hajizadeh E, Asghari M. Statistical methods and analyses in health and biosciences a research methodological approach. Tehran: Jahade Daneshgahi.2011.

[49] Vakili MM, Jahangiri N. Content validity and reliability of the measurement tools in educational, behavioral, and health sciences research. J Med Educ Dev. 2018; 10: 106–118.

[50] Mohammadbeigi A, Mohammadsalehi N, Aligol M. Validity and reliability of the instruments and types of measurments in health applied researches. JRUMS. 2015; 13: 1153–1170.

[51] Torkashvand F, Asadpor M, Rezaeian M. Validity and Reliability of Methadone Maintenance Therapy Abstinence Orientation Scale in Iran: A Short Report. JRUMS. 2015;14: 611–620.

[52] Kline R. Data preparation and psychometrics review. Principles and practice of structural equation modeling. 4th ed. New York, NY: Guilford. 2016.

[53] Vinzi VE, Chin WW, Henseler J, Wang H. Handbook of partial least squares. Springer. 2010.

[54] Fornell C, Larcker DF. Evaluating structural equation models with unobservable variables and measurement error. Journal of marketing research. 1981; 18: 39–50. doi: 10.2307/3151312

[55] Cleland V, Timperio A, Sharman MJ, Dollman J. Test‐retest reliability of a self‐reported physical activity environment instrument for use in rural settings. Aust J Rural Health. 2020;28(2):168–79. doi: 10.1111/ajr.12625 32390206

[56] Lenz E.R. Measurement in nursing and health research. Springer publishing company. 2010.

[57] Terwee CB, Bot SD, de Boer MR, van der Windt DA, Knol DL, Dekker J, et al. Quality criteria were proposed for measurement properties of health status questionnaires. J Clin Epidemiol. 2007; 60: 34–42. doi: 10.1016/j.jclinepi.2006.03.012 17161752

[58] Hair JF Jr, Hult GT, Ringle CM, Sarstedt M, Danks NP, Ray S. Partial least squares structural equation modeling (PLS-SEM) using R: A workbook. Springer Nature. 2021. doi: 10.1007/978-3-030-80519-7

[59] Brown JD. Likert items and scales of measurement. Statistics. 2011; 15: 10–14.

[60] Sangoseni O, Hellman M, Hill C. Development and validation of a questionnaire to assess the effect of online learning on behaviors, attitudes, and clinical practices of physical therapists in the United States regarding evidenced-based clinical practice. IJAHSP. 2013; 11: 7. doi: 10.46743/1540-580X/2013.1439

[61] Hoyle RH. Handbook of structural equation modeling. Second Edition, Guilford press. 2012.

[62] Vinzi VE, Chin WW, Henseler J, Wang H. Handbook of partial least squares: Concepts, methods and applications. Heidelberg, Dordrecht, London, New York. Springer. 2010.

[63] Beckett C, Eriksson L, Johansson E, Wikström C. Multivariate data analysis(MVDA). Pharmaceutical Quality by Design: A Practical Approach. John Wiley & Sons Ltd.2018. doi: 10.1002/9781118895238.ch8

[64] Neukrug E.S. and Fawcett R.C. Essentials of Testing and Assessment: A Practical Guide for Counselors, Social Workers, and Psychologists, Enhanced. Cengage Learning. 2019.

[65] Harrison CJ, Sidey-Gibbons CJ. Modern Psychometric Measurement and Computerized Adaptive Testing, in Handbook of Quality of Life in Cancer. Springer. 2022.

[66] Buchholz I, Janssen MF, Kohlmann T, Feng YS. A systematic review of studies comparing the measurement properties of the three-level and five-level versions of the EQ-5D. Pharmacoeconomics. 2018; 36: 645–661. doi: 10.1007/s40273-018-0642-5 29572719 PMC5954044

[67] Fleiss J.L., Levin B., and Paik M.C. Statistical methods for rates and proportions. john wiley & sons. 2013.

